# Geophysical insights into groundwater aquifer characterization in a geologically complex region: a case study from the Meki–Alemtena area, central Ethiopia

**DOI:** 10.1038/s41598-026-44448-x

**Published:** 2026-03-17

**Authors:** Ephrem Alemu Mehammed, Mebatseyon Shawel Bamnew, Tilahun Azagegn Tafere, Murtessa Teganu Feyisa, Yahya Ali Abdulkadir

**Affiliations:** 1 Physics Department, College of Natural and Computational Science, Haramaya University, P.O. Box: 138, Dire Dawa, Ethiopia; 2 School of Earth Science, Addis Ababa University, P.O. Box: 1176, Addis Ababa, Ethiopia; 3 Department of Applied Geology, School of Applied Natural Science, Adama Science and Technology University, P.O. Box: 1888, Adama, Ethiopia

**Keywords:** Dar Zarrouk Parameters, Groundwater potential, Magnetic survey, Transmissivity, Vertical Electrical Sounding, Environmental sciences, Hydrology, Solid Earth sciences

## Abstract

This study aimed to map geological structures and characterize groundwater aquifers in the Meki–Alemtena area of the East Shewa Zone, Ethiopia. Sixteen Vertical Electrical sounding data were collected to reveal five to seven subsurface layers across the profiles, with resistivity values indicating lithologic units such as alluvial deposits, fractured ignimbrite, and rhyolite, forming aquifers with thicknesses of 9 to 76 m. Areas with low resistivity are found to correspond with groundwater-saturated zones, particularly in the northwestern part of the study area. Aquifer characterization using Dar Zarrouk Parameters identifies three primary water-bearing zones: freshwater, brackish, and saline aquifers. High longitudinal conductance (≥ 6 Ω^−1^) and transmissivity values in the northwestern section indicate the highest groundwater potential, while hydraulic conductivity analysis suggests efficient water flow through this zone. Magnetic data further highlight structural influences on groundwater flow, with high magnetic anomalies in the south-central and northwestern areas associated with faulted and fractured zones that act as conduits for groundwater. The integration of these methods offers a comprehensive assessment of groundwater resources, establishing a framework for targeted extraction and sustainable management in the Rift Valley’s water-scarce regions.

## Introduction

**Fig. 1:**
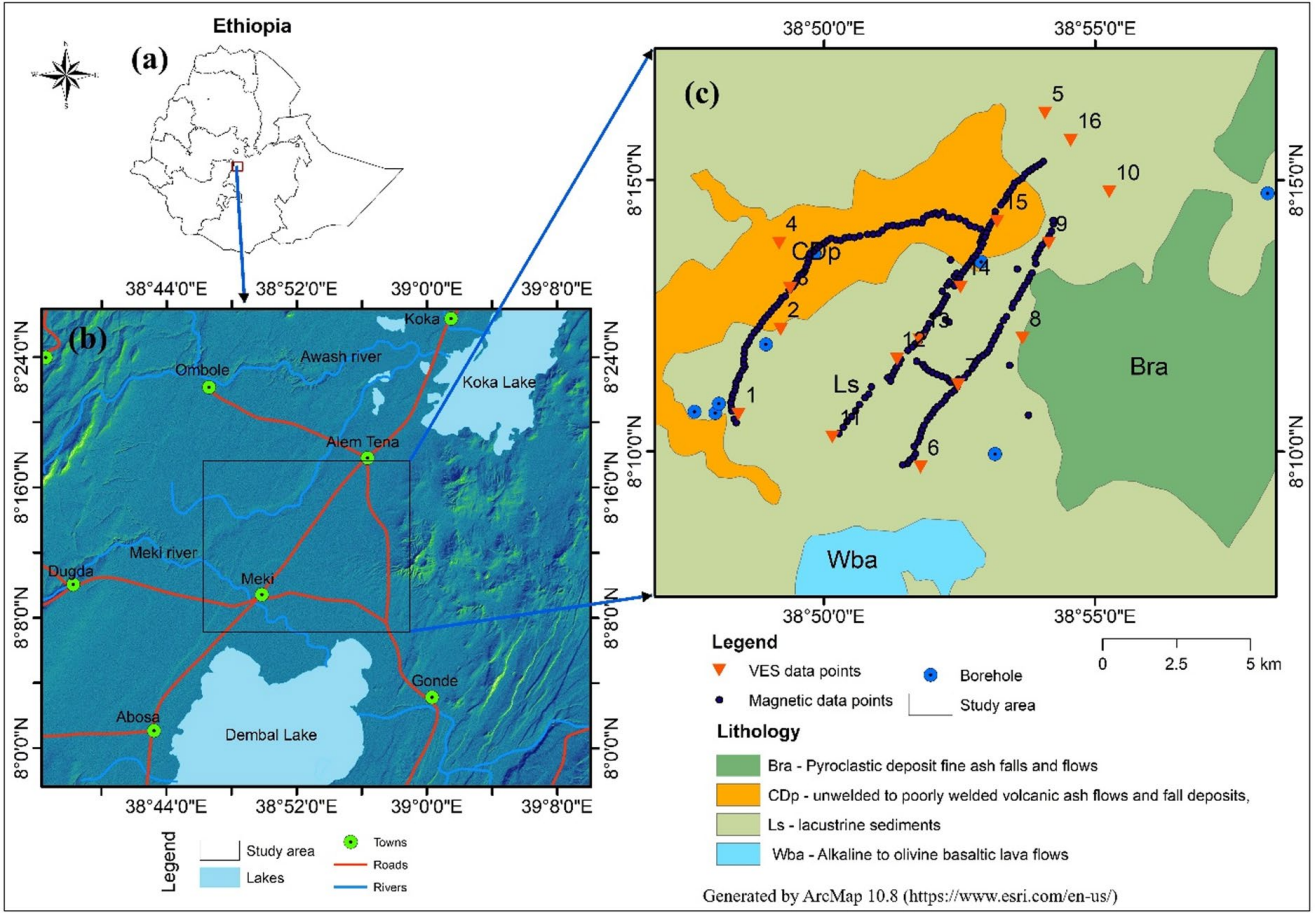
Location of the study area, (**a**) map of Ethiopia, (**b**) a location map, and (**c**) a geological map with a VES and magnetic data points.

Groundwater remains one of the most dependable sources of potable water and agricultural supply, particularly in regions with seasonal surface water scarcity^1^. In the Ethiopian Rift and other volcanic terrains of East Africa, groundwater plays a crucial role in sustaining domestic, industrial, and irrigation needs^2,3^. However, the growing imbalance between water demand and available safe supplies driven by rapid population expansion and agricultural intensification has enhanced the need for accurate aquifer characterization to ensure sustainable management^4^. The Meki-Alemtena area, located in central Ethiopian rift (Fig. 1a), is a case in point where population growth and economic activities have led to increasing water demand in recent years, yet well yields and water quality vary strongly because of the complex subsurface structure and variable lithology.

Groundwater management requires accurate knowledge of various surface and subsurface factors such as geology, physiography, and rainfall^5,6^. Recent reviews in the Ethiopian Rift emphasize the dominant role of geological structures in controlling groundwater in volcanic terrains and the need for more integrated geophysical-hydrogeological workflows in data-scarce settings^7,8^. Understanding the geometry and hydraulic behavior of these formations is therefore essential for sustainable groundwater development and for mitigating the large spatial variability in well productivity frequently observed in rifted volcanic settings^9^.

Previous studies in the Ethiopian Rift show that effective groundwater management depends on thorough aquifer characterization based on hydrogeological and geophysical studies^10,11^. Numerous studies that employ integrated geophysical methods, mainly electrical resistivity sounding (VES) and magnetic surveys, have been conducted to assess and characterize groundwater potential zones^12–14^. This integrated approach is essential for accurately assessing groundwater potential especially in regions with complex hydrogeological settings. Several studies have also extensively shown the importance of application of VES data analysis techniques, specifically the Dar Zarrouk parameters, for characterization of aquifers^15^.

At regional scales, groundwater potential mapping has been done using Remote Sensing based multi-criteria evaluation, Analytical Hierarchy Process models, and more recently machine-learning and artificial-intelligence algorithms^16,17^. While these data-driven frameworks are effective for reconnaissance zoning but do not provide the vertical stratification and physical parameters required to quantify aquifer properties. By contrast, physics-based geophysical surveys, particularly VES and magnetic measurements can directly estimate resistivity contrasts, identify weathered and fractured zones, and reveal structural controls on groundwater flow^18^. When integrated with borehole information, they allow quantitative interpretation of subsurface architecture in areas where borehole data are sparse.

The present work addresses this gap by applying an integrated VES, magnetics, and borehole approach for assessing groundwater potential especially in regions with complex hydrogeological settings^19,20^. This study also uses Dar Zarrouk parameters to evaluate important hydrogeological characteristics, such as hydraulic conductivity and transmissivity, in order to better describe the aquifer system. By integrating these characteristics with the combined interpretation of VES and magnetic data sets, the study provides a more precise understanding of underlying lithology and its hydrological features. This will make it possible to identify groundwater-bearing structures with greater accuracy. As a result, it adds new methodological and regional insights to Ethiopian groundwater exploration. Addressing this question through a geophysical framework not only supports local water planning but also contributes to the international discourse on groundwater dynamics in tectonically active volcanic regions. Methodologically, this study introduces a transferable workflow linking Dar Zarrouk parameters with magnetic lineaments to delineate structurally controlled groundwater pathways in volcanic rocks. The aim is to provide insights to guide groundwater resource management in the Meki–Alemtena area, supporting both current and future water needs. It also provides a new hydro-structural perspective for groundwater development in faulted volcanic terrains, with implications that extend beyond local management to similar rift environments^21^.

### Description of the study area

The study area is located at the hydrological boundary between the Awash River Basin to the north and the Rift Valley Lakes Basin to the south, within the central segment of the Main Ethiopian Rift, between the towns of Meki and Alemtena (Fig. 1(a, b)). Geographically it is located between a latitude of 8° 06’ 0” to 8° 18’ 00” N, and longitude of 38° 46’ 0” to 39° 00’ 00” E covering a total area of 450.3 km² (Fig. 1c). The study area has an elevation range of 1,603 to 2,167 m asl, and is characterized by a semiarid to arid climate^22^. The region exhibits a bimodal rainfall pattern, with the main rainy season occurring from June to September, and a smaller rainfall between March and May^23^, with a total annual rainfall ranging from 700 to 950 mm^24^. The average annual temperature is approximately 23 °C, and the major land cover in the area includes cropland, shrubland, and scattered forest, with increasing human pressure resulting from agricultural expansion and urban growth^24^.

### Geology

The geological history of the Main Ethiopian Rift (MER) is characterized by extensive volcanism, sedimentation, and tectonic activity during the Cenozoic Era^25^. Tertiary volcanic rocks dominate the geological sections along the rift borders, while the crystalline basement is occasionally found covered by the Tertiary volcanics and Mesozoic sedimentary rocks^26^. The dominant geological units in the MER include Quaternary volcanic rocks such as ignimbrite, rhyolite, scoria, basalt flows, lacustrine deposits, and alluvial sediments. The Meki-Alemtena area, specifically, is primarily characterized by Quaternary volcanic rocks, including ignimbrites, rhyolites, and basalts, along with significant deposits of lacustrine and alluvial sediments. Lacustrine deposits, primarily composed of silt and clay-sized material with a small amount of volcanoclastic sediment and tuff, cover a significant portion of the area, particularly around Lake Ziway. These deposits are considered Pleistocene to Recent in age^27^. Alluvial sediments are found in the valley plains, primarily composed of sandy clay and silty clay with gravel in the lower bed, and are known for their high permeability, contributing to local shallow aquifers. The fault systems that dominate the region are mainly the NNE–SSW-trending Wonji Fault Belt (WFB) and E–W trending transfer faults, which govern groundwater occurrence and flow^28,29^. It is known for its large grabens and a significant offset between the rift floor and the plateau^26,30^. These faults and associated structures significantly influence groundwater flow patterns and aquifer development in the region. Fault intersections and fracture zones enhance recharge and form high-yield aquifers, whereas unfractured rhyolitic domes restrict permeability.

### Hydrogeology and water demand

Groundwater is the primary water source for domestic, industrial, and agricultural uses in this region, as surface water is seasonal and frequently saline. Recent estimates indicate that domestic water demand has risen by over 40% in the past two decades due to urbanization around Meki town and irrigated horticulture expansion^31^. However, borehole yields vary widely from less than 2 L/s in compacted rhyolite to over 10 L/s in fractured ignimbrite reflecting heterogeneous aquifer conditions^32^. This necessitates detailed subsurface mapping to guide sustainable abstraction and avoid overexploitation of the limited fresh groundwater zones. The region’s complex geological history, shaped by intense tectonic activity and volcanism, has resulted in a dynamic landscape characterized by extensive faulting, volcanic activity, and intricate groundwater flow patterns^33,34^. Understanding this geological context is crucial for effectively managing groundwater resources in this water-scarce region^31^.

## Materials and methods

This study employed a combination of geophysical methods, namely vertical electrical sounding (VES), and magnetic surveys, to map the subsurface geological structures and characterize groundwater aquifers in the Meki-Alemtena area. The transects used for the geophysical investigations are shown in Fig. 1c. Several steps were taken during data acquisition and processing to guarantee the quality of the field VES and magnetic data and reduce the impact of noises, such as power lines, roads, and metallic infrastructure. In order to minimize coupling with adjacent conductive structures, VES stations were placed away from significant sources of electrical interference during fieldwork, and electrode orientations were changed as needed. The contact resistance was examined to guarantee steady measurements before to recording each sounding, and further readings were obtained to verify data consistency. During data processing, noisy curves were carefully examined and filtered, and any anomalous data segments that were most likely caused by interference were removed or corrected. The same is true for magnetic data points; in order to verify the accuracy of our measurement, noisy magnetic data points were eliminated prior to any magnetic correction.

### Resistivity methods

The most effective and important method of groundwater exploration is the resistivity technique of geophysical prospecting^35^. This technique has been successfully applied to groundwater studies due to its effectiveness and non-destructive behavior in creating subsurface images. Depending on the local area’s resistivity readings, the resistivity method can be used to assess the aquifer’s depth, stratigraphy, and water quality^36^. Depth measurement and horizontal profiling (lateral resistivity variation) are two main ways of using electrode arrays^37^. The Vertical Electrical soundings (VES) is one of the electrical resistivity approaches that can be used in various investigations in which the center and orientation of the potential configuration remain constant. To obtain the apparent resistivity values, the resistance measurements from the resistivity meter were multiplied by the corresponding geometric factor as shown in Eqs. (1) and (2). In this study, 16 VES measurements were conducted using a PASI 16 GL Earth Resistivity Meter paired with a P100-3 Energizer, utilizing the Schlumberger array configuration with a maximum half-electrode spacing (AB/2) of 500 m (Fig. 1c). In this configuration, the potential electrodes are next to one another, while the current electrodes are symmetrically spaced at progressively longer intervals around a central point^38^.

1 $${\rho}_{a}=K\times\frac{\varDelta V}{I}$$

2 $$K=\pi\left(\frac{{\left(\frac{AB}{2}\right)}^{2}-{\left(\frac{MN}{2}\right)}^{2}}{MN}\right)$$

where${\rho}_{a}$ is the apparent resistivity, $k$is the geometric factor, $\varDelta V$ is the measured potential difference, $I$ is the injected current, AB/2 is half the spacing between current electrode, and MN/2 is half the spacing between potential electrode.

Two key parameters in electrical prospecting, are the Dar Zarrouk parameters of^39^, which are described by the longitudinal unit conductance (C) and the transverse unit resistance (R)^40^. These variables correspond to various combinations of each geoelectric layer’s thickness and resistivity, as shown in Eqs. (3) and (4). These parameters are frequently related to the Hydraulic conductivity (K) and Transmissivity (T) of an aquifer as they can indicate the aquifer’s ability to defend itself from contamination and its permeability, respectively. The analytical relationship between transmissivity and longitudinal conductance on one hand, and transmissivity and transverse resistance on the other, was derived by ^41^ from Darcy’s equation and the differential form of Ohm’s law, as shown in Eqs. (5) and (6).

3 $$R=\rho h$$

4 $$C=h/\rho$$

5 $$T=K\sigma C$$

6 $$T=K\sigma R$$

where *R* is the transverse resistance, $\rho$ is resistivity of the layer in ohm meters, h is the layer thickness in meter, *T* is the transmissivity, K is the hydraulic conductivity and *C* is the longitudinal conductance.

At each of the 16 VES locations, several metrics, including hydraulic conductivity, transmissivity, and coefficient of anisotropy, were determined in order to explain the hydraulic characteristics of the aquifers and the groundwater potential. Using these approach, the aquifer’s hydraulic conductivity (K) and Transmissivity (T) were estimated by using Eqs. (7) and (8) respectively as given by^38,41^. The electrical conductivity or resistivity properties of a material is measured by the coefficient of anisotropy$\left(\lambda\right)$. It is calculated as the ratio of maximum to minimum electrical resistivity, as given in Eq. (9)^42^.

7 $$K=368.4{\rho}^{-0.93283}$$

8 $$T=Kh$$

9 $$\lambda=\sqrt{\frac{\rho t}{\rho l}}$$

Where K is hydraulic conductivity, $\rho$ is an aquifer resistivity, T is Transmissivity, h is an aquifer thickness, $\lambda$ is coefficient of anisotropy, $\rho t$ transverse resistivity, $\rho l$ is longitudinal resistivity.

### Magnetic survey

Magnetic survey comprises determining the direction, gradient, and strength of the magnetic field as well as interpreting changes in these parameters throughout the survey area^43^. The technique is frequently used for subsurface research to identify geological formations that could store groundwater in the studied area^44^. The most common use of magnetic data in groundwater studies is to map the depth of magnetic bedrock and to identify faults and geological connections^45^. A Proton Precession Magnetometer was used to conduct the magnetic survey, resulting in 225 data points collected across the study area. Qualitative interpretations of magnetic anomaly indicate the existence or absence of subsurface geological features /buried targets and is converted to a constrained geological mode^46^. Correcting the magnetic data is crucial to cancel out just those sources of magnetic anomalies resulting from the effects of the subsurface on the observations^35^. The effects of daily changes of magnetic anomalies are corrected by using diurnal variation adjustment, given on Eq. (10), where base values account only for time-dependent fluctuations^35^.

10 $$\delta d=\left(\frac{BS2-BS1}{T2-T1}\right)\left(Ti-T1\right)$$

Were, $\delta$d is diurnal correction, BS1 is reading of base station at first time, BS2 is reading of base station at second time, T1 is observation time of first base station, T2 is observation time of second base station, and Ti is observation time of each measurement.

An impact of the geomagnetic reference field on the survey data is removed by the geomagnetic adjustment. Survey data at any specific area can be fixed by deducting the theoretical value, obtained from IGRF, from the actual value (Bob). As a result, Total magnetic field anomaly ($\varDelta B$), which is produced by removing the diurnal correction ($\delta B$D) and IGRF correction (Bth) from the total magnetic field is given by Eq. (11). The processed magnetic data were then used to generate various magnetic maps, including total anomaly, analytical signal, vertical and horizontal derivatives, and reduced to the equator maps.

11 $$\varDelta B=BT-\delta B D-Bth$$

Where, BT is the total magnetic field, $\delta BD$ is diurnal correction and Bth is an IGRF correction.

The Analytical Signal (AS) is a mathematical technique that extracts the amplitude and phase information from the original magnetic data. This technique highlights shallow sources including the edges and boundaries of magnetic anomalies^47^ and but not the dip information. The AS is commonly used in magnetic interpretation to locate the anomalies directly over their sources and is calculated by Eq. (12).

12 $$AS=\sqrt{{\left(\frac{\partial\varDelta M}{\partial x}\right)}^{2}+{\left(\frac{\partial\varDelta M}{\partial Y}\right)}^{2}+{\left(\frac{\partial\varDelta M}{\partial z}\right)}^{2}}$$

Where AS is amplitude of analytic signal and $\varDelta M$is observed total magnetic anomaly.

## Result and discussion

### 1D modeling of VES data

Apparent resistivity curves from 16 VES stations were inverted using a layered-earth, 1-D forward model with iterative least-squares optimization. Win-resist software was used to create input models for VES interpretation, iteratively refined by comparing model outputs with field data to achieve an acceptable fit. For each station, we iterated until either the normalized RMS misfit stabilized, or parameter updates fell below a small threshold. The final models are those that simultaneously minimize misfit and remain hydrogeologically reasonable across adjacent stations. It yields RMS error ranging between 1.2% and 2.5%, which indicates the quality of the match between model and field data.

**Fig. 2:**
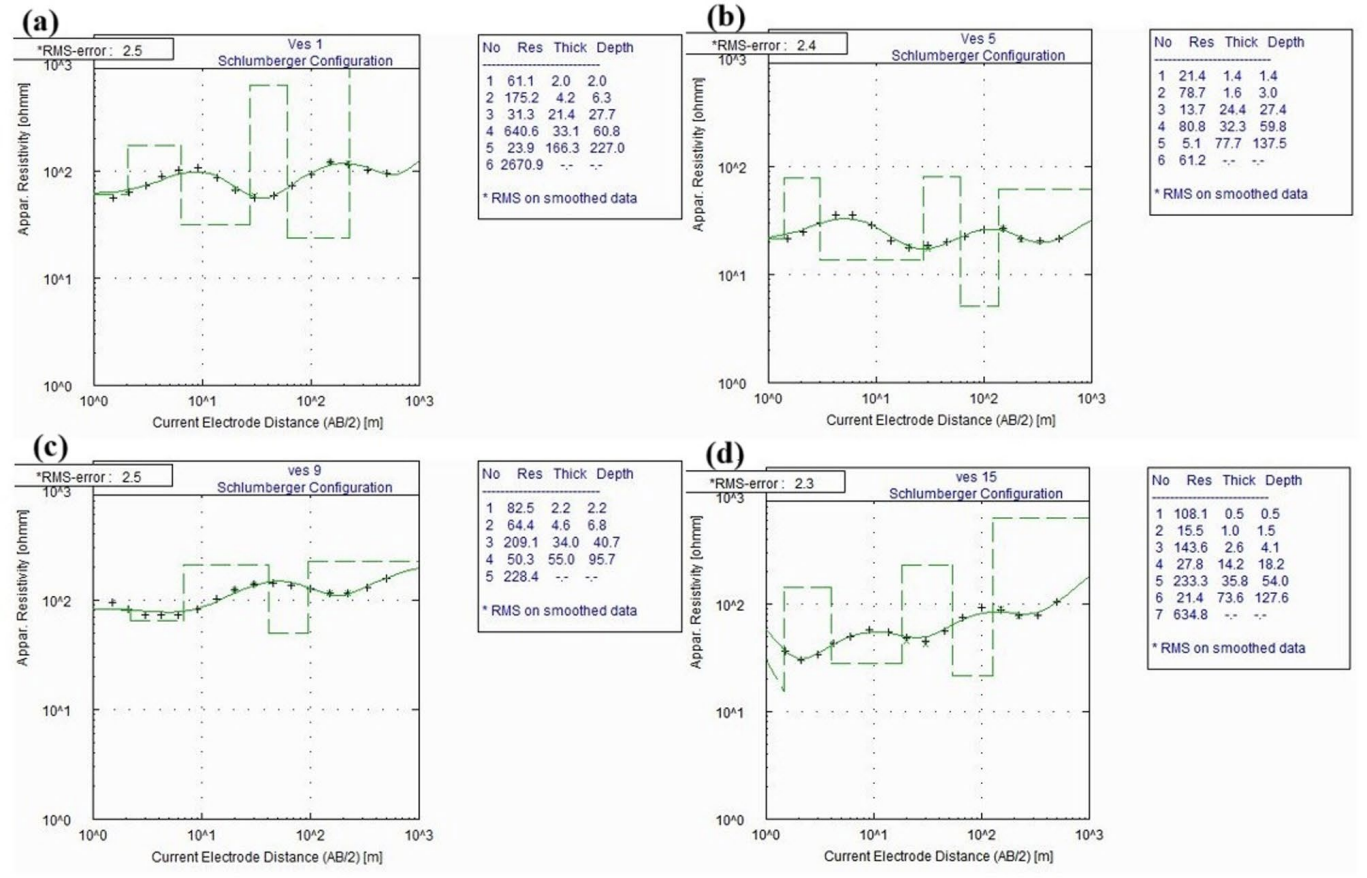
Some VES curves, (**a**) VES 1, (**b**) VES 5, (**c**) VES 9, (**d**) VES 15, and their interpretation.

The interpreted geoelectric models reveal five to seven subsurface layers corresponding to distinct lithological units as shown in Fig. 2(a–d). Table 1 shows typology of interpreted VES data in the study area. The dominant VES curves type includes KHKH (50%), HKHKH (18.75%), HKHK (12.5%), HKH (12.5%) and AKQH (6.25%) (Table 1). The dominant KHKH curve type suggests alternating conductive and resistive layers of volcanic sequences interbedded with heterogeneous subsurface with possible multiple aquifer zones separated by impermeable units^48,49^. Borehole data near VES 2, 7, and 14 validate these interpretations, confirming the correlation between resistivity layering and lithologic boundaries. These results demonstrate the resistivity structure-based delineation of aquifer geometry across the Meki–Alemtena watershed divide, providing the foundation for hydro-structural interpretation developed in later sections.

**Table 1:** Typology of Sounding curves in the study area.

VES Stations	Curve nature	Curve types
VES 1	$\rho1<\rho2>\rho3<\rho4>\rho5<\rho6$	KHKH
VES 2	$\rho1>\rho2<\rho3>\rho4<\rho5>\rho6$	HKHK
VES 3	$\rho1>\rho2<\rho3>\rho4<\rho5>\rho6$	HKHK
VES 4	$\rho1<\rho2>\rho3<\rho4>\rho5<\rho6$	KHKH
VES 5	$\rho1<\rho2>\rho3<\rho4>\rho5<\rho6$	KHKH
VES 6	$\rho1<\rho2>\rho3<\rho4>\rho5<\rho6$	KHKH
VES 7	$\rho1<\rho2>\rho3<\rho4>\rho5<\rho6$	KHKH
VES 8	$\rho1>\rho2<\rho3>\rho4<\rho5$	HKH
VES 9	$\rho1>\rho2<\rho3>\rho4<\rho5$	HKH
VES 10	$\rho1<\rho2<\rho3>\rho4>\rho5<\rho6$	AKQH
VES 11	$\rho1<\rho2>\rho3<\rho4>\rho5<\rho6$	KHKH
VES 12	$\rho1>\rho2<\rho3>\rho4<\rho5>\rho6<\rho7$	HKHKH
VES 13	$\rho1<\rho2>\rho3<\rho4>\rho5<\rho6$	KHKH
VES 14	$\rho1<\rho2>\rho3<\rho4>\rho5<\rho6$	KHKH
VES 15	$\rho1>\rho2<\rho3>\rho4<\rho5>\rho6<\rho7$	HKHKH
VES 16	$\rho1>\rho2<\rho3>\rho4<\rho5>\rho6<\rho7$	HKHKH

### Geoelectric profile sections

#### Geoelectric section along profile 1

**Fig. 3:**
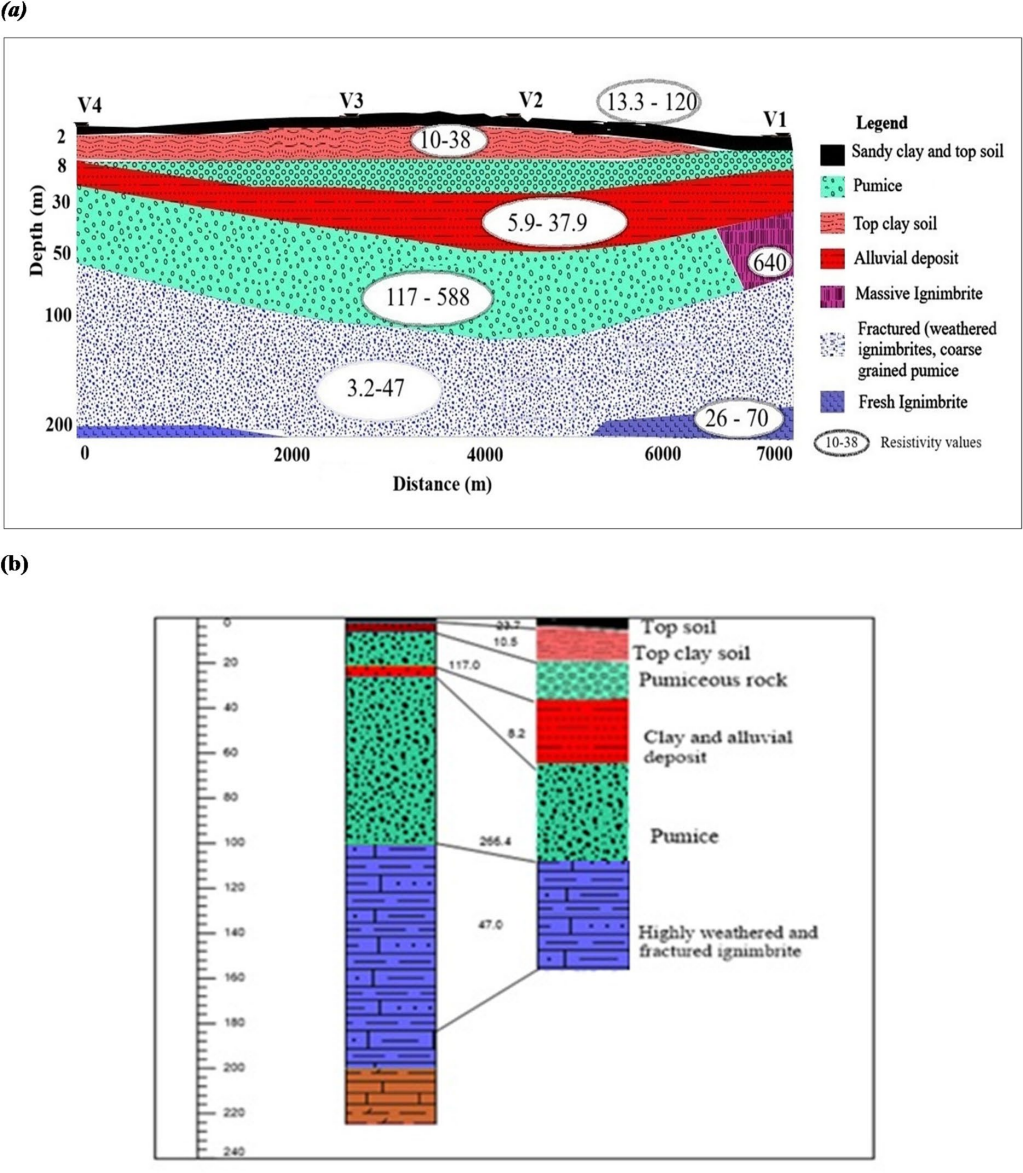
**(a)** Geoelectric section along profile 1. **(b).** VES 2 and lithology log at Sera Wakele Borehole.

The subsurface stratigraphy reveals six distinct geoelectric layers (Fig. 3a). The top layer, with resistivity and thickness ranging from 13.3 to 120 Ωm and 1.3–2.4 m, respectively, is characterized by a thin surficial deposit and interpreted as topsoil. The second layer, with resistivity of 10.5–37.9 Ωm and thickness of 2.8–5.6 m, is interpreted as a mixture of clay and sand. The third and fifth layers exhibit higher resistivity, ranging from 117 to 538 Ωm with a thickness of 3–7 m, and are interpreted as pumice deposits, ignimbrite, or rhyolite. The fourth layer, with low resistivity values of 5.9–31 Ωm and thickness between 9 and 27 m, is inferred to be an alluvial clay deposit. The sixth layer is highly weathered, fractured and saturated, indicating the main aquifer, with low resistivity values of 3.2–47 Ωm. Comparison of the VES results with the closest borehole data from the Meki area (Fig. 3b) supports this stratigraphic interpretation, demonstrating consistency between geophysical data and observed geological units.

### Geoelectric section along profile 2

**Fig. 4:**
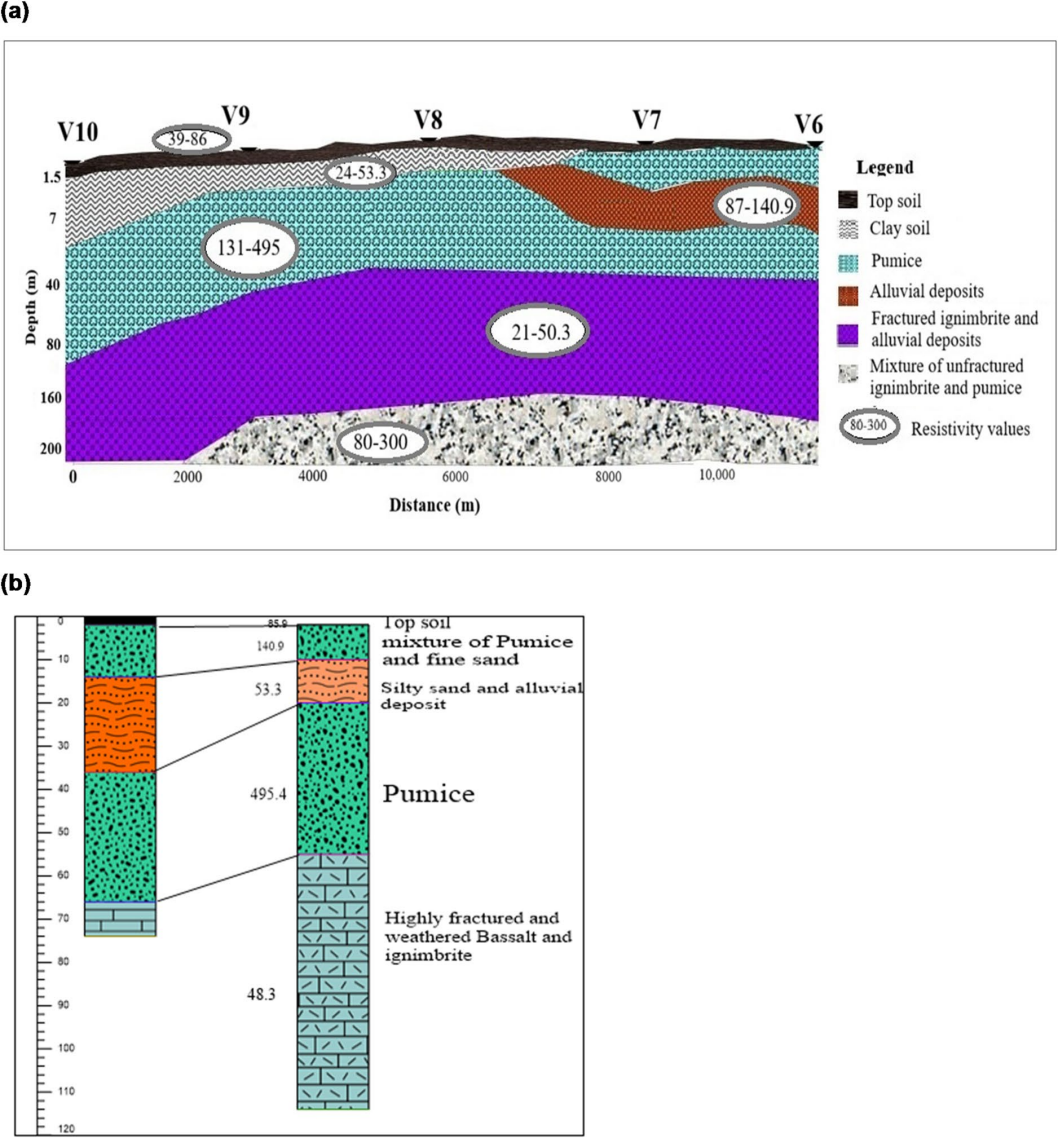
**(a)** Geoelectric section along profile two. **(b).** VES 7 and lithology log of Tuchi Sumeya Borehole.

The geoelectric section along Profile 2 (Fig. 4a), constructed from VES–6 to VES–10, provides valuable insights into the subsurface lithology, supported by borehole data from the nearby Tuchi Sumeya borehole near VES–7 and VES–8 (Fig. 4a). The top layer, comprising clay soil and fine-grained sand, has resistivities of 39–95 Ωm and a thickness of 1.5–2.2 m, and is identified as topsoil. The second layer, with resistivities of 24–53.3 Ωm and 87–141 Ωm and a thickness of 1.6–5.6 m, corresponds to pumice, alluvial deposits, or clay. The third layer, interpreted as moderately permeable pumice mixed with sand, exhibits resistivities of 131.9–495 Ωm and thicknesses of 34–38.6 m. The fourth layer, extending to depths of up to 250 m beneath VES–10, shows low resistivities (21–50.3 Ωm) and considerable thicknesses (15.7–114.4 m), suggesting highly fractured ignimbrite and alluvial deposits with high porosity and permeability, indicative of potential groundwater-bearing zones. The deepest layer, observed beneath VES points 6 to 9, shows resistivity values of 80–299 Ωm, attributed to unfractured ignimbrite and pumice. Figure 4b illustrates the correlation between VES–7, and the lithological log of the Tuchi Sumeya borehole. The lithological logs align closely with the geoelectric section, validating the interpretation of the subsurface materials. Minor discrepancies observed may be attributed to the lateral distance between the borehole locations and the VES survey points. Overall, integrating geoelectric section and borehole data strengthens the subsurface interpretation and highlights the groundwater potential of the fractured, highly porous ignimbrite zones.

### Geoelectric section along profile 3

**Fig. 5:**
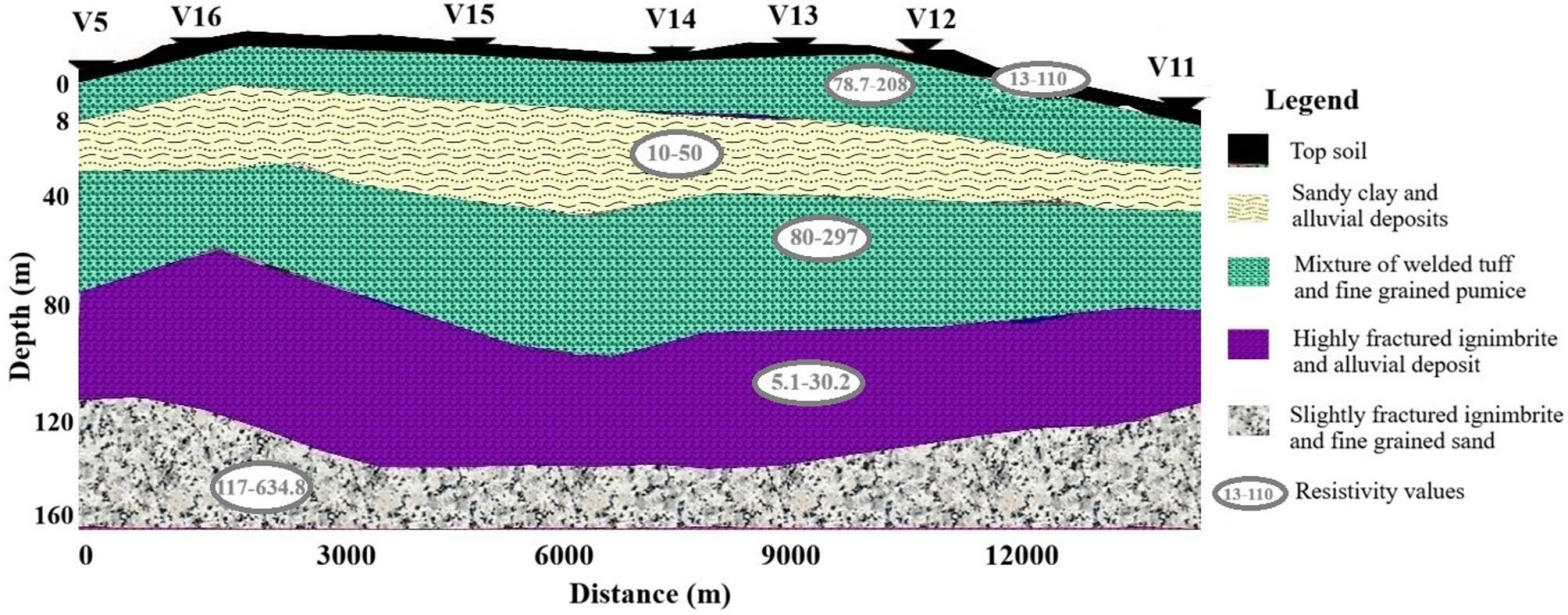
Geoelectric cross section of Profile three.

The geoelectric section along Profile 3 (Fig. 5) was constructed using seven VES points (VES–11 to VES–16 and VES–5). Borehole data from the nearby Berta borehole (near VES–14 and VES–15) aided in lithological identification and depth correlation. The top layer, with resistivities of 14.6–108 Ωm and thicknesses of 1.5–2.7 m, represents topsoil. The second layer, identified as pumice, shows resistivities of 78.7–237 Ωm and thicknesses of 2.5–6.21 m. The third layer, likely sandy clay and alluvial deposits, has resistivities of 10.9–67.5 Ωm and thicknesses of 7.9–23.7 m. The fourth layer, with resistivities of 80.8–297 Ωm and thicknesses of 25–51 m, suggests coarse-grained pumice and weathered ignimbrite. The fifth layer, with low resistivities of 5.1–30.2 Ωm and significant thicknesses of 52–83.3 m, is interpreted as fractured ignimbrite with conductive materials, indicating high groundwater potential. The deepest, highly resistive layer (117–634.8 Ωm) is attributed to slightly fractured ignimbrite mixed with pumice.

### Integrated view of the three profiles

The three geoelectric profiles (Fig, 3a, 4a, and 5) collectively reveal a layered subsurface structure characterized by topsoil, pumice, alluvial deposits, fractured ignimbrite, and a highly resistive base layer. Each profile highlights varying aquifer potential, with significant groundwater prospects identified in heavily fractured ignimbrite and pumice-rich layers. Borehole correlation (Sera Wakele, and Tuchi Sumeya wells) indicates that these horizons coincide with productive water-bearing units, whereas compact rhyolite beneath them forms a hydraulic barrier. Consistent lithological correlation between VES data and borehole logs strengthens the reliability of the interpretations. The spatial continuity of these conductive zones suggests a semi-confined aquifer system with variable transmissivity controlled by fracture density. Such alternation between permeable and impermeable layers is a hallmark of volcanic rift aquifers, consistent with findings from the Belesa catchments^20^.

### Aquifer characterization using dar zarrouk parameter

The Dar Zarrouk parameters derived from the VES-inverted layer resistivities, and thicknesses (Table 2) provide a quantitative basis for evaluating aquifer hydraulic behavior across the Meki–Alemtena region. These parameters allow classification of aquifer salinity, protective capacity, and groundwater potential^40,50^. The Dar Zarrouk parameters are a key indicator in groundwater modeling and management, as they reflect an aquifer’s hydrogeological properties by combining transmissivity and storage coefficients, thus measuring an aquifer’s water storage capacity.

**Table 2:** Summary statistics of the VES variables for the entire data set considered in the Dar Zarrouk parameter.

VES No	Resistivity of Aquifer (Ωm)	Aquifers thickness (h)	Depth to aquifer (H)	Longitudinal Conductivity (C)	Transverse Resistance (*R*)	Longitudinal Resistivity (ρl)	Hydraulic Conductivity (K)	Transmissivity (T)	Coefficient of Anisotropy ( $\boldsymbol{\lambda}$)	Protective Capacity
1	23.90	166.00	227.00	6.95	3967.40	32.68	19.08	3167.28	0.65	2.10
2	47.00	108.60	173.00	2.31	5104.20	74.87	10.15	1102.29	0.29	0.06
3	3.20	37.50	150.00	11.72	120.00	12.80	124.48	4668	1.56	0.15
4	9.40	126.00	181.90	13.40	1184.40	13.57	45.56	5740.56	1.52	0.03
5	5.10	77.70	137.50	15.24	396.27	9.03	80.59	6261.843	2.25	0.26
6	21.20	116.20	184.80	5.48	2463.44	33.72	21.33	2478.546	0.63	0.22
7	48.30	59.20	114.00	1.23	2859.36	93.01	9.99	591.408	0.24	0.01
8	21.20	25.70	77.60	1.21	544.84	64.01	21.33	548.181	0.33	0.10
9	50.30	55.00	95.70	1.09	2766.50	87.52	9.53	524.15	0.25	0.01
10	30.30	46.20	160.00	1.52	1399.86	104.94	15.29	706.398	0.21	0.43
11	18.80	51.30	112.50	2.73	964.44	41.23	23.86	1224.018	0.51	0.08
12	17.30	83.70	150.30	4.84	1448.01	31.07	25.79	2158.623	0.68	0.19
13	23.50	62.00	122.90	2.64	1457.00	45.58	19.38	1201.56	0.46	0.12
14	30.20	56.90	123.40	1.88	1718.38	69.74	15.34	872.846	0.31	0.04
15	21.40	73.60	127.60	3.44	1575.04	37.10	21.15	1556.64	0.57	0.15
16	13.50	67.30	110.20	4.99	908.55	22.11	32.50	2187.25	0.95	0.11

### Longitudinal conductance (C)

**Fig. 6:**
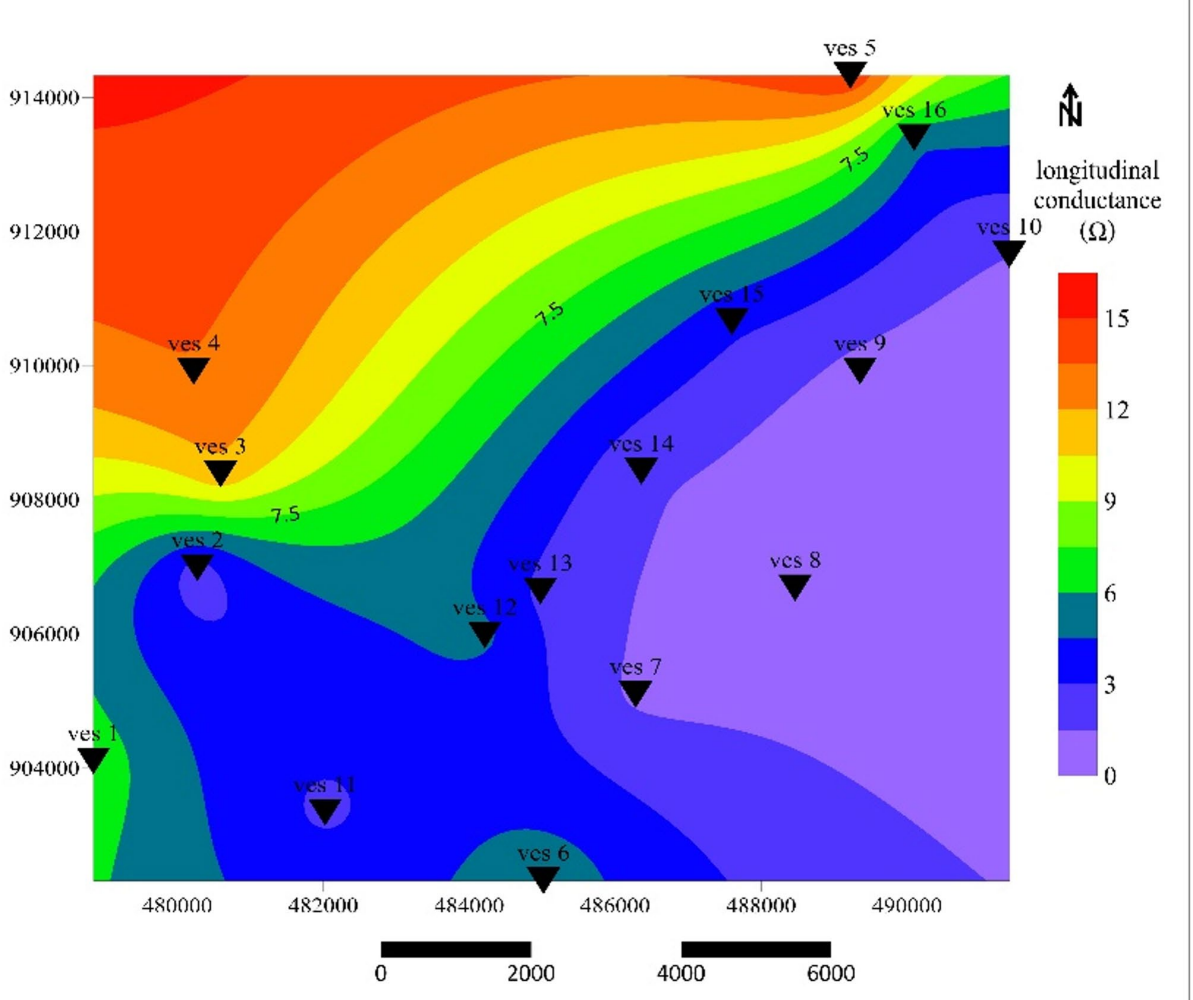
Longitudinal conductance contour map.

Longitudinal conductance (C) values in the study area range from 1.09 to 15.24 Ω⁻¹, indicating significant spatial variability in aquifer thickness and clay content. The longitudinal conductance contour map (Fig. 6) delineates three aquifer zones: Freshwater (VES 7, 8, 9, 10, 13, 14), Brackish water (VES 6, 11, 12, 15, 16), and Saline water (VES 1, 3, 5). High C values occur primarily corresponding to zones of thick, conductive material interpreted as alluvial or clay-rich deposits. These areas represent saline aquifers, consistent with low longitudinal resistivity and low transverse resistance later discussed. While lower C values in the southeastern sector reflect thinner, more resistive layers characteristic of freshwater-bearing fractured ignimbrite^51^. This zonation agrees with global interpretations of C as an indicator of aquifer salinity and transmissive capacity^21,50^. Freshwater zones generally align with the elevated southeastern terrain, supporting the influence of topography and lithologic variability on groundwater recharge.

### Transverse resistance (R)

**Fig. 7:**
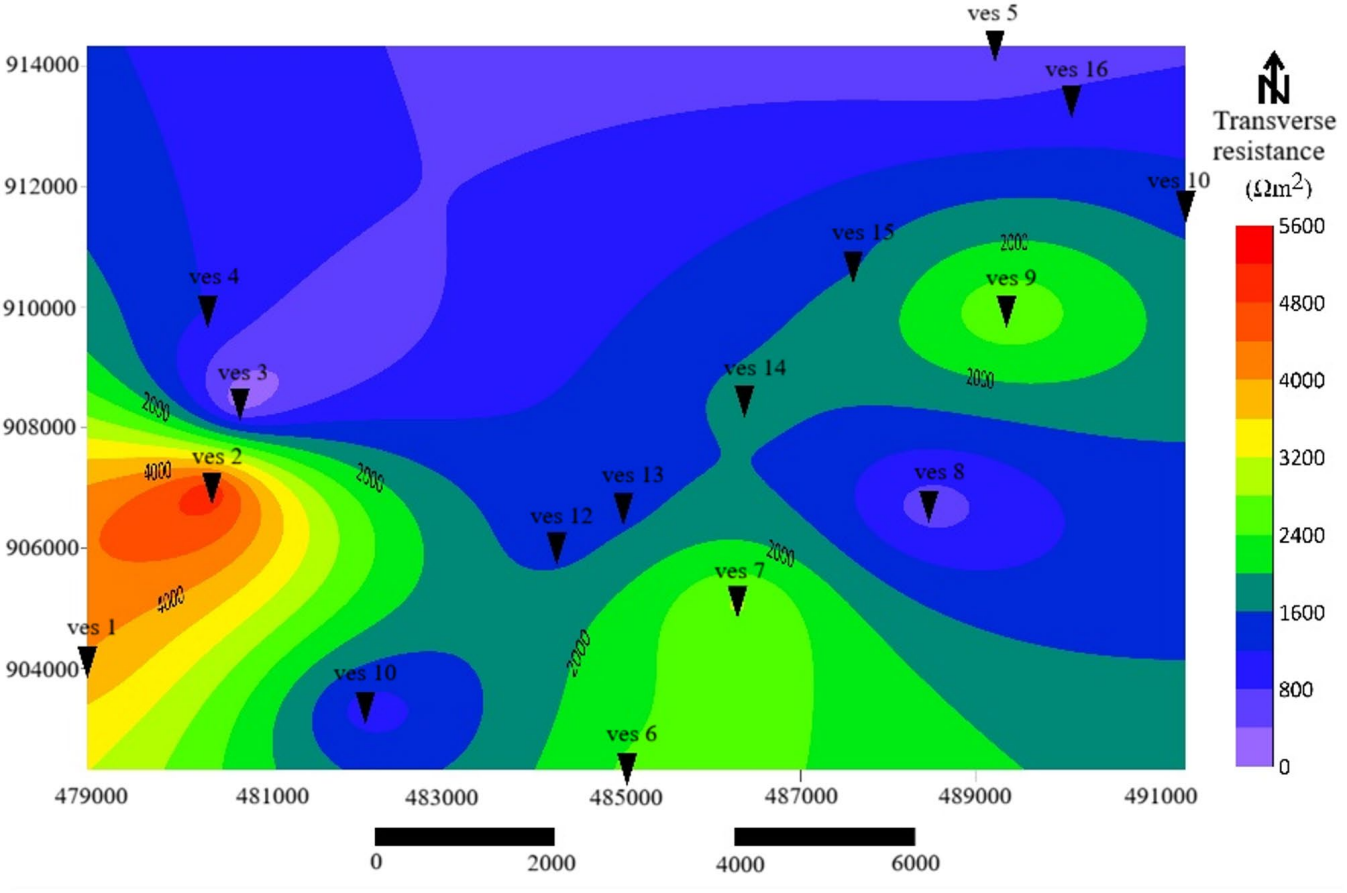
Traverse Resistance contour map.

Transverse resistance values range from 120 to 5,600 Ω·m², helping differentiate aquifer quality and hydraulic potential. The transverse resistance map (Fig. 7) delineates three aquifer types: High transverse resistance (> 2,400 Ω·m²) occurs in the eastern and southeastern parts (VES 2, 7, 9, 10), signifying thick, resistive formations such as fractured ignimbrite and pumice. These units form the freshwater aquifer system with relatively high permeability. Moderate R (1,200–2,400 Ω·m²) in the central zone corresponds to brackish aquifers, representing transitional lithologies. Low R (< 1,200 Ω·m²) in the northern sector (VES 3, 4, 5, 8, 11) confirms the presence of saline, clay-rich, or saturated alluvial materials. Notably, zones of high transmissivity combined with low transverse resistance highlight promising targets for groundwater development. These areas should be prioritized for groundwater development, particularly in regions where water demand is critical. The combined C–R relationships match classifications used in similar volcanic terrains^52^. Low R, together with high C, is particularly diagnostic of saline zones with limited protective capacity.

### Longitudinal resistivity $\left(\rho l\right)$

**Fig. 8:**
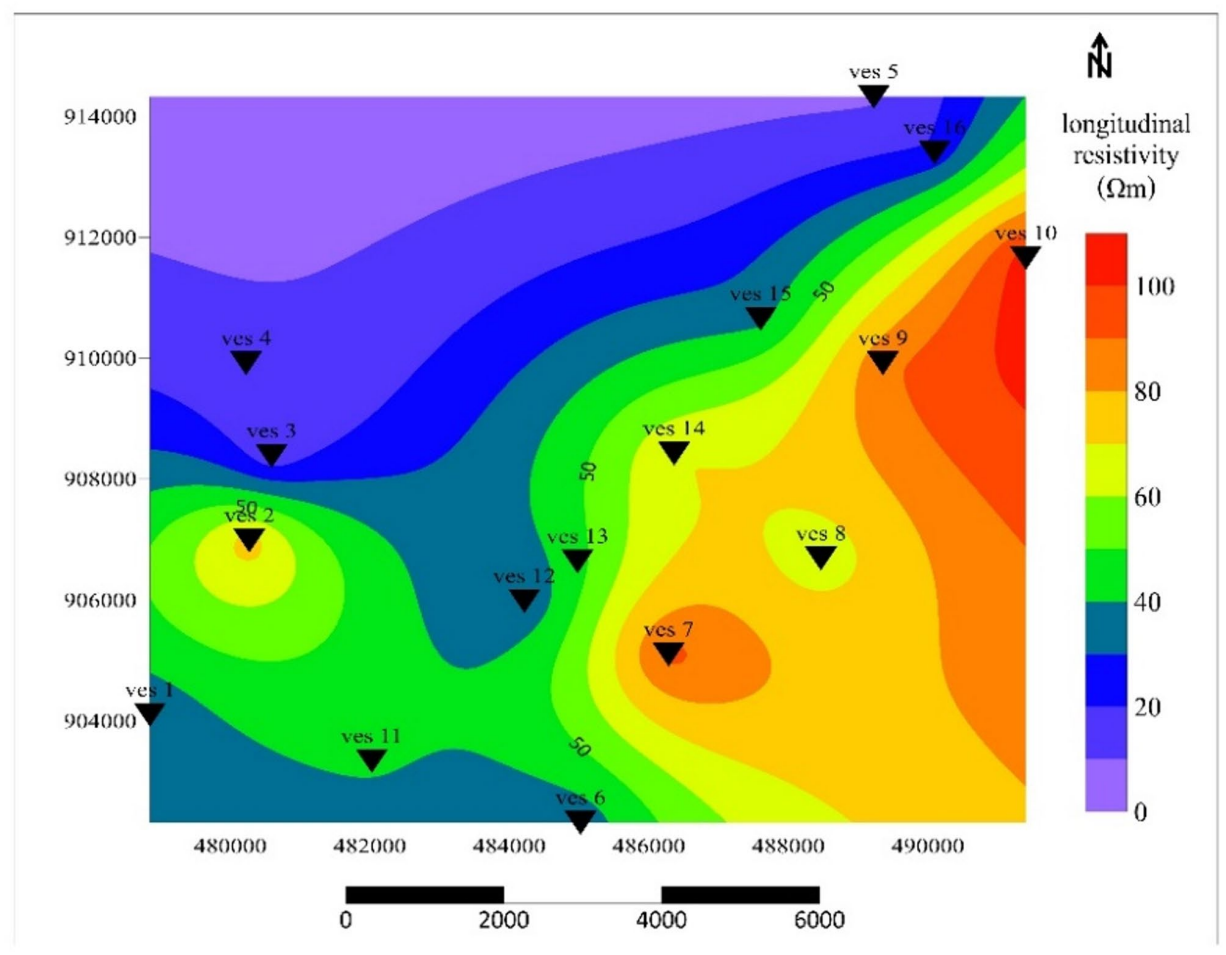
Longitudinal Resistivity contour map.

Longitudinal resistivity (ρ*l*), values in the study area vary from 9 to 105 Ωm, enabling the separation of aquifer types by water quality as shown in Fig. 8. Saline water aquifers (0–32 Ω·m) exhibit low resistivity due to high salinity; brackish water aquifers (32–72 Ω·m) have intermediate resistivity typical of transitional zones; and freshwater aquifers (> 72 Ω·m) display high resistivity reflecting low ion content. These resistivity thresholds are consistent with global DC-resistivity interpretations for volcanic and sedimentary aquifers^39,41^. The findings emphasize the utility of longitudinal resistivity as a diagnostic tool for hydrogeological studies, guiding groundwater management and development efforts by identifying zones with varying water quality. Freshwater zones align with permeable pumice-ignimbrite assemblages, whereas low longitudinal resistivity in the north indicates high ionic concentration.

### Hydraulic conductivity (K)

**Fig. 9:**
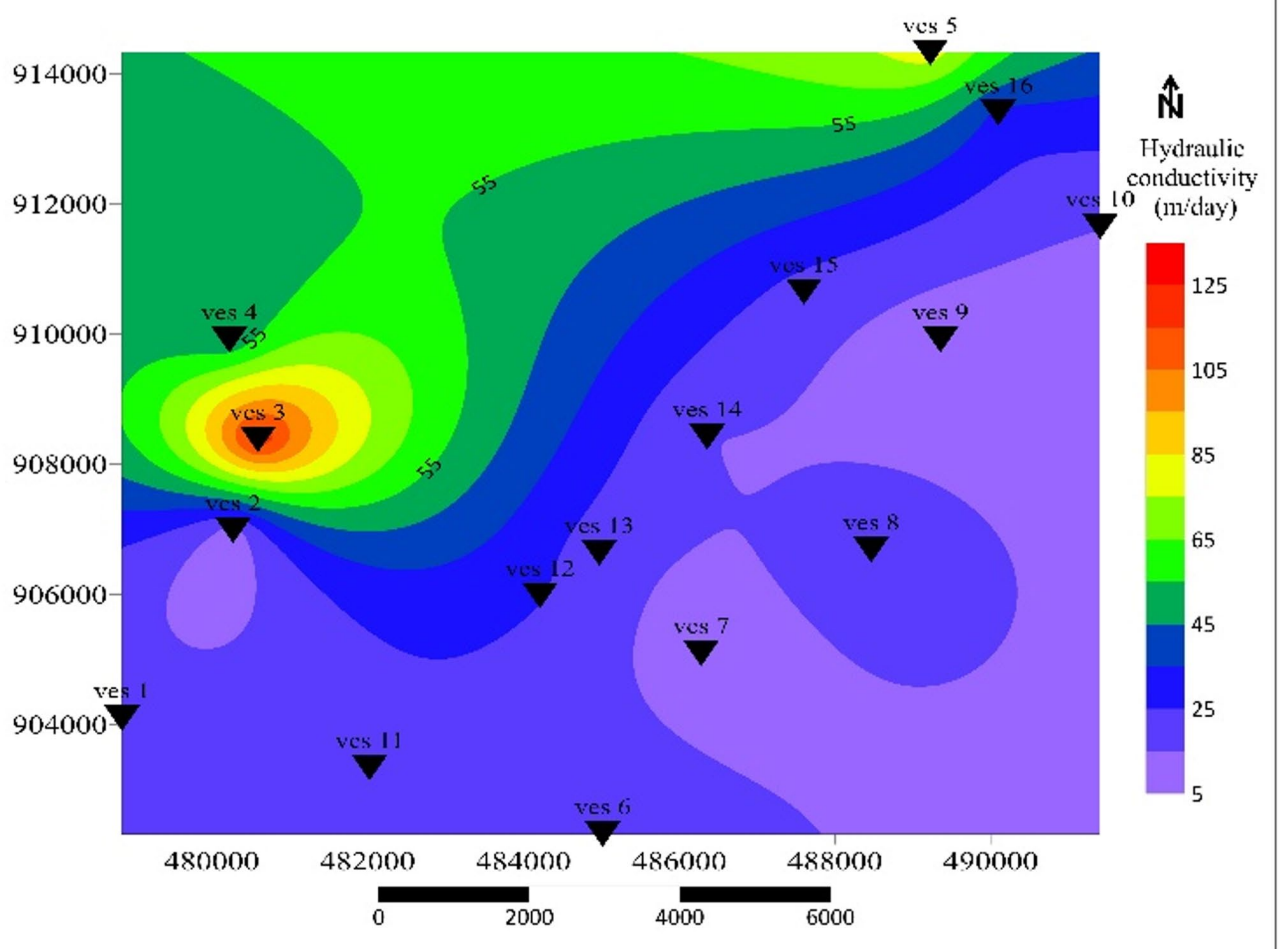
Distribution of aquifer hydraulic conductivity contour map.

Hydraulic conductivity (K) of the aquifer, varying from 5 to 135 m/day, indicating substantial spatial variation in aquifer permeability as presented in Fig. 9. Low K (5–50 m/day) covers much of the central and southern terrain, reflecting semi-confined aquifers in layered volcanic deposits with clay intercalations. Moderate K (50–100 m/day) defines transitional zones where pumice and ignimbrite are moderately fractured. High K (100–135 m/day) is concentrated in the northern sector, indicating a highly fractured ignimbrite that enhances groundwater flow. This low value highlights the complexity of groundwater flow due to the geological constraints of the semiconfined aquifers^53^, whereas high K value enhanced groundwater movement and a potentially valuable zone for water extraction. These values closely align with published K ranges for similar volcanic aquifers in the Ethiopian Rift^54^.

### Transmissivity (T)

Transmissivity in the study area ranges from 524 to 6262 m²/day (Table 2), directly reflecting the combined effects of hydraulic conductivity and saturated thickness. High transmissivity (T > 2200 m²/day) in the north and northwest corresponds to fractured ignimbrite with significant saturated thickness up to 126 m. This result agrees with^32^, and it indicates significant groundwater potential and high permeability for fluid movement^55^. Moderate transmissivity (1200–2200 m²/day) appears in the central and southeastern zones, suggesting moderately productive aquifers. Low transmissivity (< 1200 m²/day) in the southern and eastern regions results from compacted pumice, tuff, and fine sediments that restrict vertical and lateral permeability. Zones of high transmissivity indicate aquifers capable of storing and transmitting significant water volumes, making them prime targets for groundwater exploration and development. A strong empirical correlation between K and T further confirms the reliability of the derived hydraulic parameters. Such relationships are consistent with volcanic aquifer studies across different areas^40,55^.

### Coefficient of Anisotropy$(\lambda$)

**Fig. 10:**
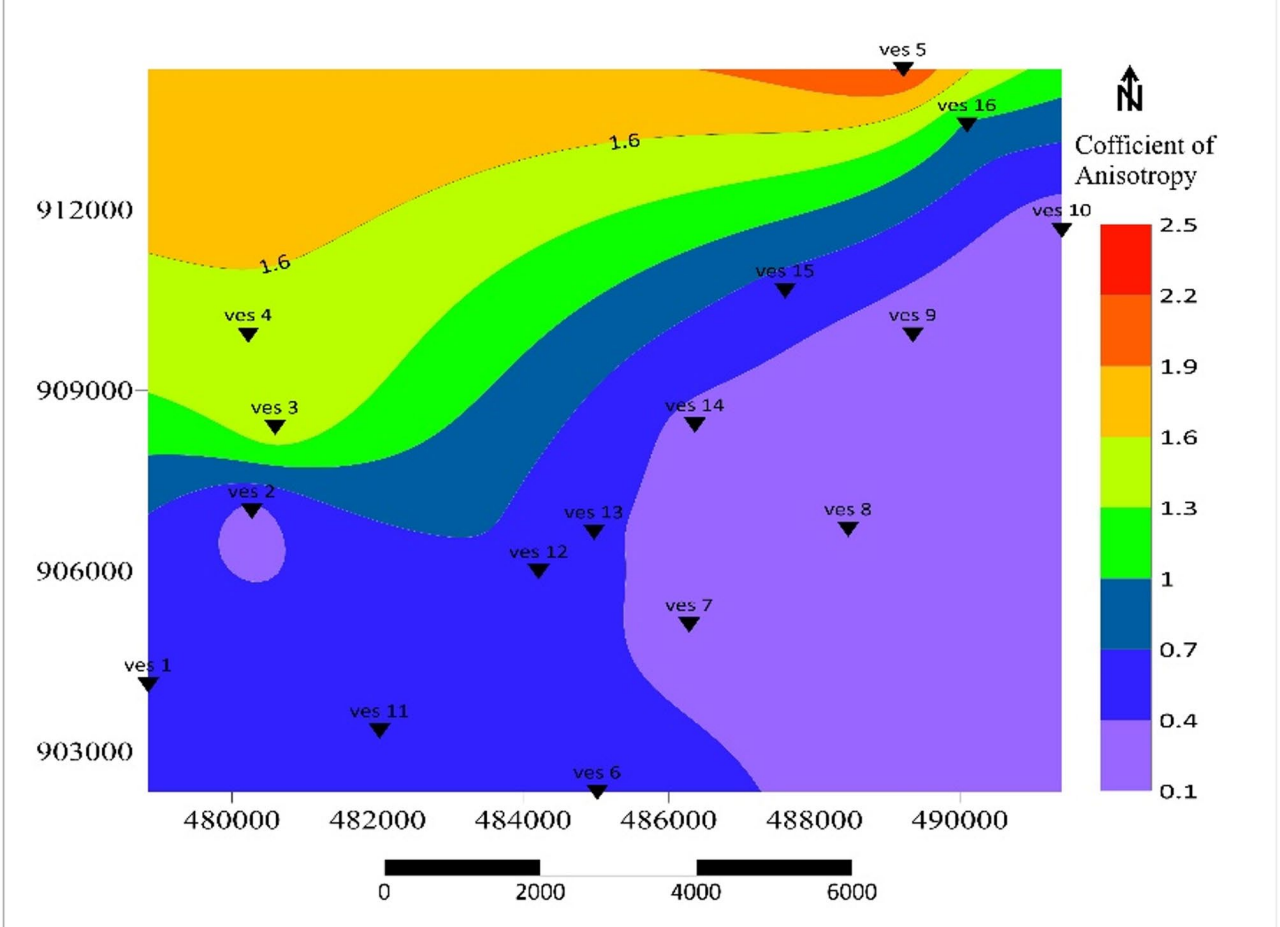
Coefficient of Anisotropy contour map.

The anisotropy (λ) values span from 0.21 to 2.25, indicating variations in subsurface heterogeneity and lateral changes in hydraulic conductivity. The anisotropic contour map (Fig. 10) provides a detailed representation of aquifer flow path. According to^56^, low anisotropy values (λ < 1.5) in eastern and northeastern regions imply hydraulically uniform, high-quality aquifers. Moderate anisotropy (1.5–2.5) marks zones of variable lithology typical of the central corridor. High Anisotropy (λ ≥ 2.5) in localized northern regions indicates subsurface heterogeneity aligned with fault-controlled hydro structural contrasts, suggesting preferential flow paths and potential barriers to groundwater movement as regions with increased compaction and rock durability tend to exhibit higher anisotropy coefficients (λ).

The boundary between high and low anisotropy values effectively reflects subsurface contacts, highlighting areas where geological units with contrasting hydraulic properties meet. This boundary is critical for understanding aquifer behavior, including flow dynamics, recharge potential, and areas of potential aquifer vulnerability. The anisotropic analysis not only aids in identifying groundwater flow paths but also provides insights into aquifer storage capacity and the influence of geological structures on groundwater movement. These interpretations follow^51,56^.

### Aquifer protective capacity

**Fig. 11:**
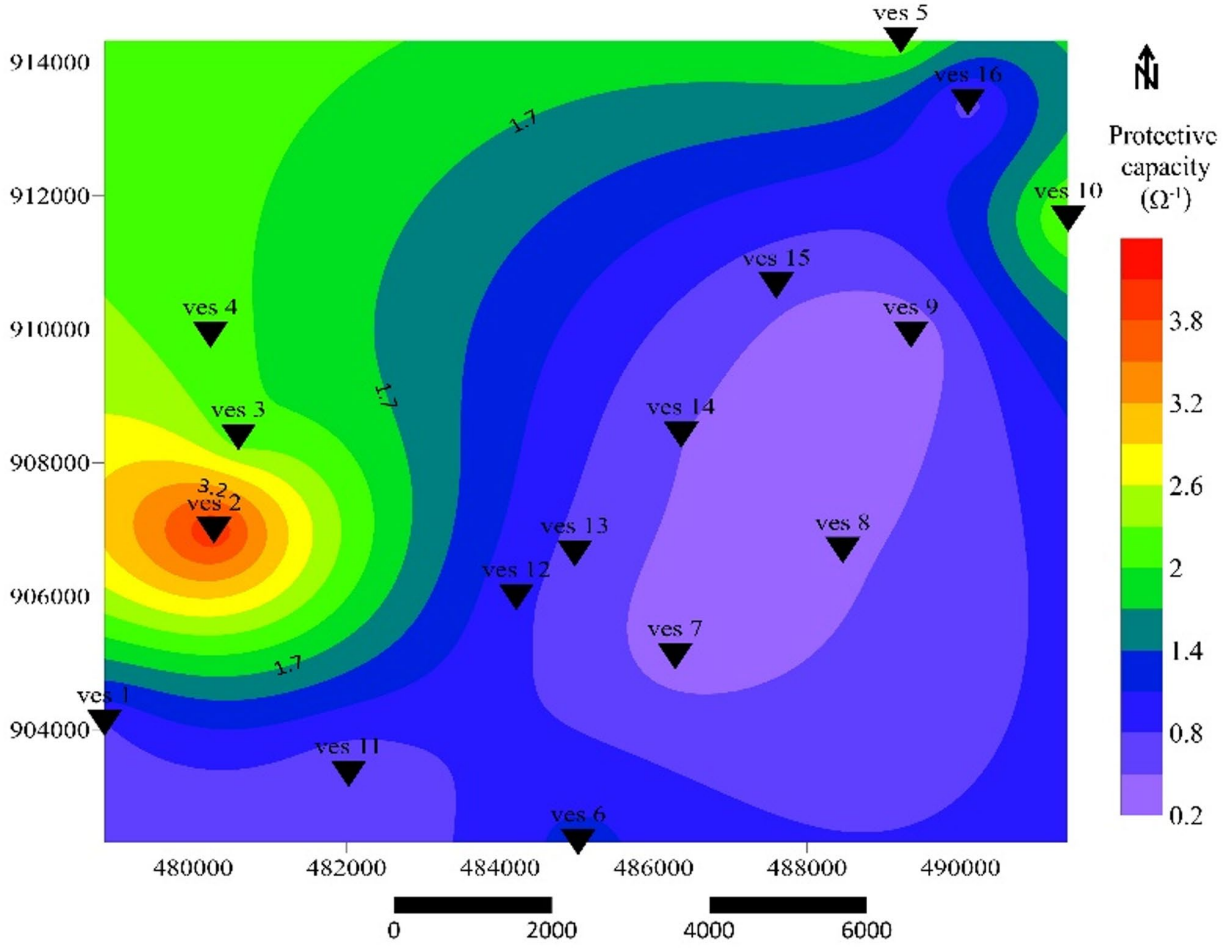
Aquifer protective capacity map.

The aquifer overburden protective capacity of the study area was evaluated following the classification of^57^ as follows: Excellent to very good protection (C > 5 Ω⁻¹) in the northwestern and northern sectors, where thick clayey overburden shields deeper aquifers. This signifies strong protection of groundwater resources. Good protection (C = 0.8–4.9 Ω⁻¹), in central corridor offering moderate resistance to contamination and Moderate to weak protection (C = 0.2–0.79 Ω⁻¹), reflecting limited protection and moderate vulnerability; and Weak (C < 0.2 Ω⁻¹), in southeastern regions, indicating vulnerability to contamination due to shallow fractured materials. The aquifer protective capacity map (Fig. 11) reveals that the study area is predominantly categorized within the moderate to good protection zones. These results correspond well with the longitudinal conductance zonation and highlight areas requiring careful groundwater development.

This distribution implies that groundwater salinity increases toward the rift axis. The pattern is analogous to the Dar Zarrouk-based aquifer zonation reported by^21,58^. High C and low R values mark thick, conductive layers capable of transmitting significant water volumes but also more vulnerable to salinization. Hydraulic conductivity (Fig. 9) and transmissivity distribution confirm that the northern and northwestern sectors exhibit the highest groundwater potential.

### Magnetic data

#### Total magnetic field anomaly

**Fig. 12:**
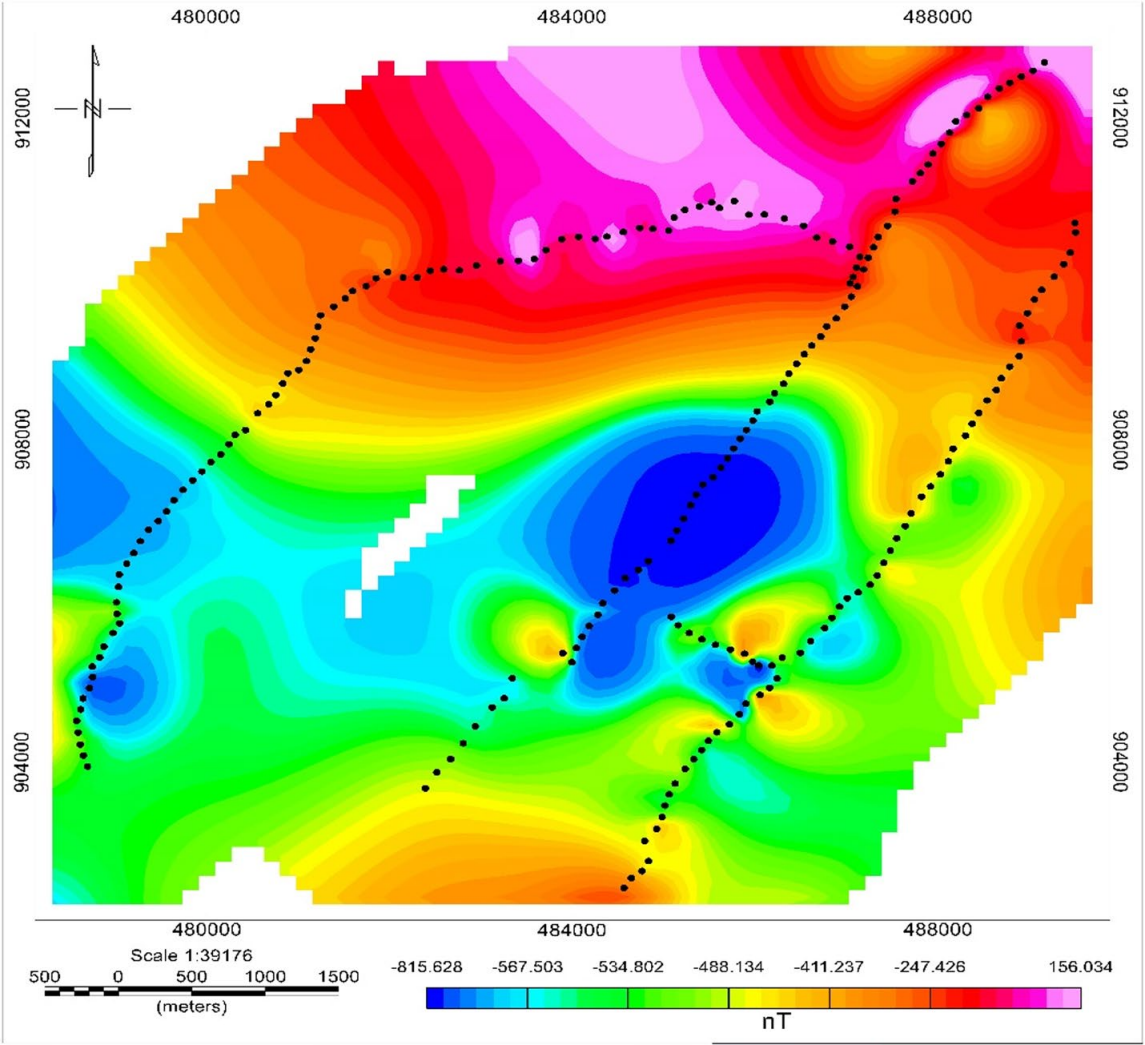
Total magnetic anomaly map of meki to alemtena area.

The Total Magnetic Anomaly (TMA) map (Fig. 12) exhibits low-to-moderate magnetic intensities across the study area, with two broad negative anomalies (–815 to 200 nT) trending east–west in the central and southwestern regions. Variations in the magnetic field reflect the underlying geological features. Most of the study areas exhibit low to moderate magnetic anomalies. Such negative anomalies typically correspond to less magnetized volcanic ash, pumice, or weathered ignimbrite, common in the upper Main Ethiopian Rift (MER). Positive high magnetic anomalies (100–166 nT) in the north and southeast likely reflect relatively unweathered ignimbrite or basaltic intrusions, consistent with their high magnetic susceptibilities. High anomaly values in the northern region may be due to mineralized silty clay and fractured ignimbrite, possibly influenced by nearby faulting and fracturing. Low anomalies in the middle and western areas are likely caused by cross ridges, while high anomalies in the southeastern region correlate with fault-like structures and volcanic ash deposits. These anomaly patterns are structurally coherent and suggest deep-seated contrasts between volcanic units and tectonic blocks typical of the MER, consistent with findings of^59^.

#### Reduction to equator

**Fig. 13:**
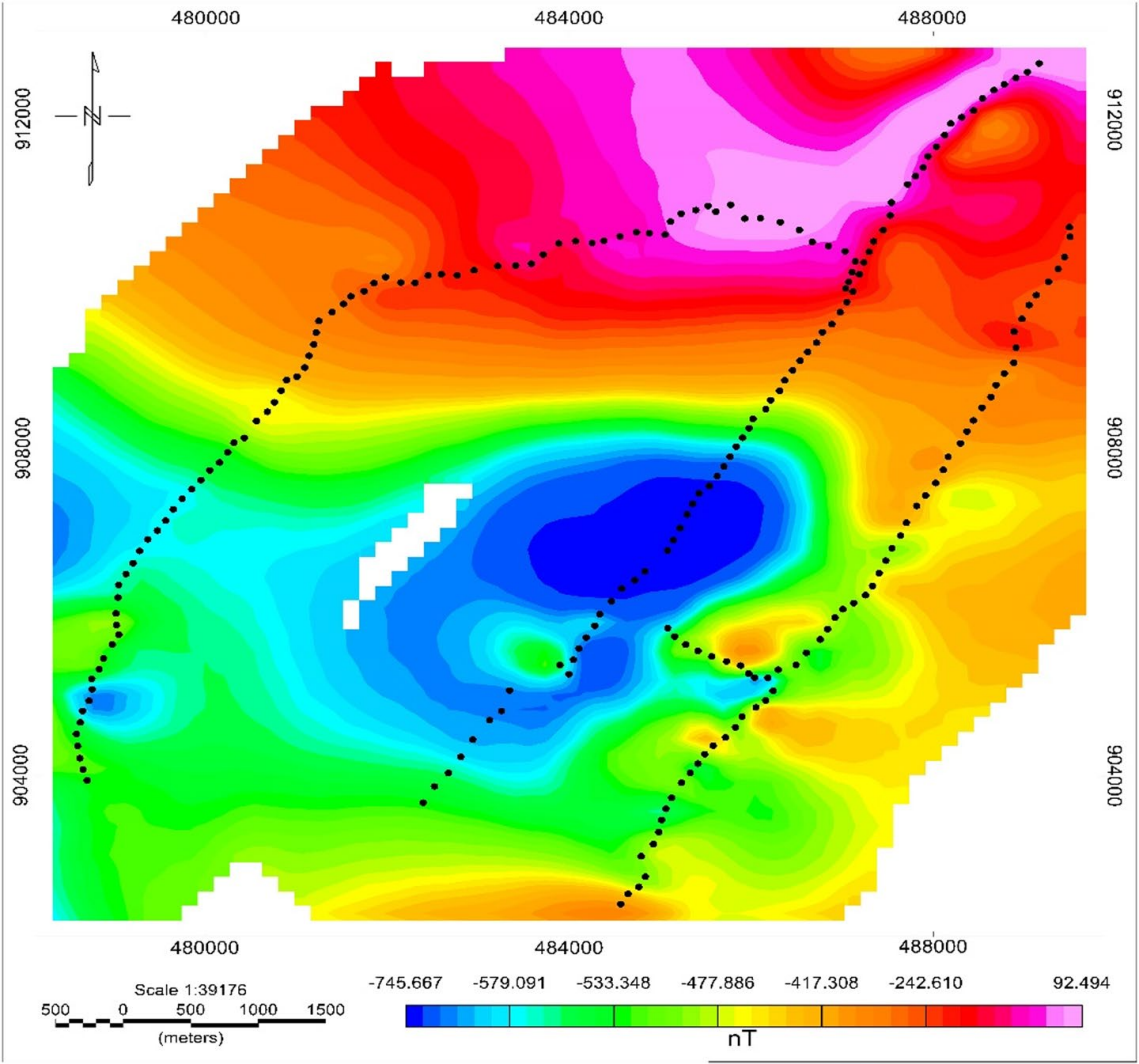
Reduced to the equator anomaly map of the area.

The RTE map (Fig. 13) centers magnetic anomalies directly above their causative bodies, improving structural boundary interpretation in low magnetic latitudes. It reveals three distinct magnetic zones, influenced by the magnetic properties of subsurface rock formations: The first zone is in the western and central regions, with very low magnetic intensity (–745 to -520 nT), reflecting thick pumice and weathered volcanic products. The second zone is found in the central corridor with moderate anomalies (-520 to -240nT), likely representing mixed lithologies (sand, silt, ignimbrite). The last zone is occupying the northern and north-central areas, strong positive anomalies (-240 to + 174 nT), associated with buried volcanic units, probable intrusive, or dense ignimbrite formations. These zones align with observed lithological transitions in VES profiles, corroborating the alternation of low and high-density volcanic materials across the basin margin.

#### Residual magnetic field anomaly

**Fig. 14:**
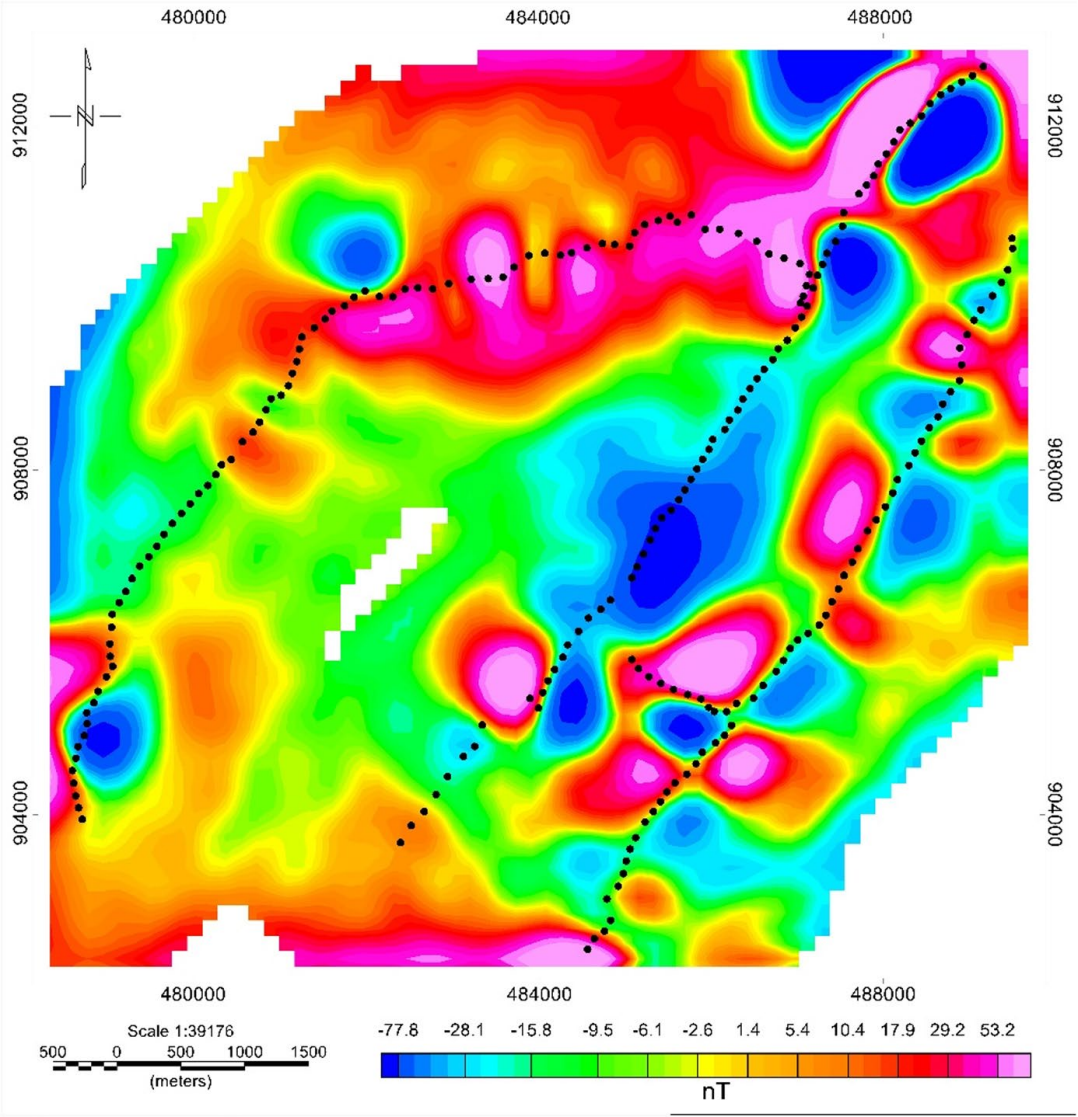
Residual Magnetic Anomaly map of Meki-Alemtena area.

After separating the regional anomaly, the residual magnetic anomaly map (Fig. 14) highlights shallow structural features dominated by elongated high-amplitude zones in the northern, eastern, and southern regions. Low residual anomalies in the central area suggest thick accumulations of non-magnetic sediments (lacustrine/alluvial deposits), consistent with low-resistivity VES layers (5–40 Ωm). This pattern likely reflects volcanic ash deposits and fragmented Quaternary sediments prevalent in the central to northern parts of the region. The pattern indicates that shallow structural heterogeneity corresponds to changes in lithology and fracturing, with potential implications for localized groundwater storage.

#### Analytic signal (AS)

**Fig. 15:**
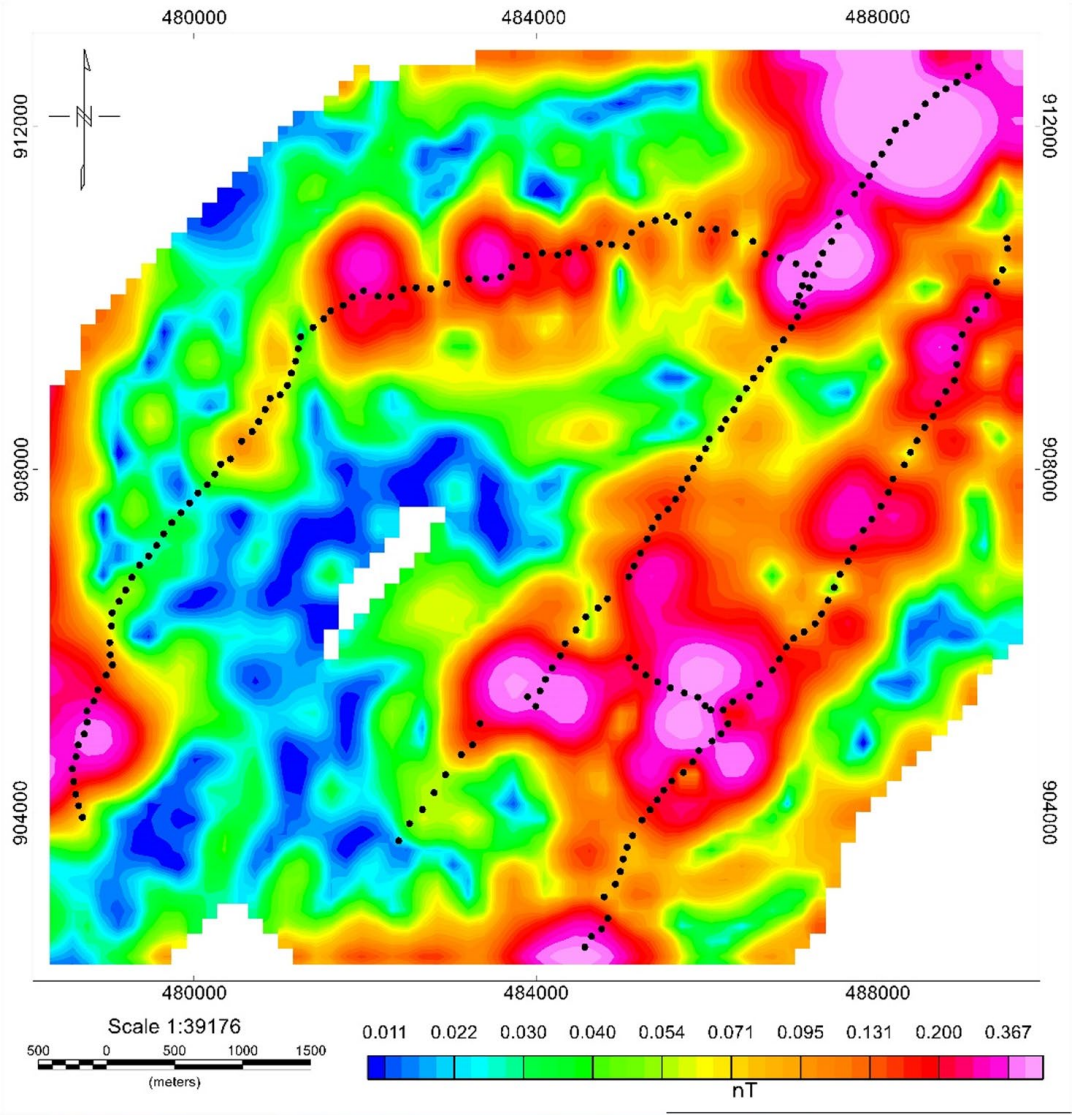
Analytic signal map of Meki-Alemtena area.

It is applied on the low pass filtered total anomaly at a cutoff wavelength of 500 m. The AS map (Fig. 15) enhances edges of magnetic bodies, with high amplitudes marking abrupt magnetization contrasts at lithologic and structural boundaries. High AS amplitudes occur in the South-central region, Southeastern corridor, and Northwestern zone. These high-amplitude bodies correspond to fault-controlled intrusive contacts and fractured volcanic units, which are common groundwater conduits in rift volcano-tectonic environments^43,47^. In the AS map displayed in (Fig. 15), the borders of the intrusive bodies and faults are clearly marked. This is influenced by the locations of the source body but not by the magnetization inclinations. The high amplitude of AS in the regions exhibits discontinuities. This discontinuity may be caused by the shearing effect. These highest positive analytic signals are aligned in the NE –SW direction, which coincides with the general structural trend of the area. The alignment of the strongest AS responses along a NE–SW trend supports the influence of the Wonji Fault Belt (WFB), a major extensional feature in the MER^28^.

#### Tilt angle derivative

**Fig. 16:**
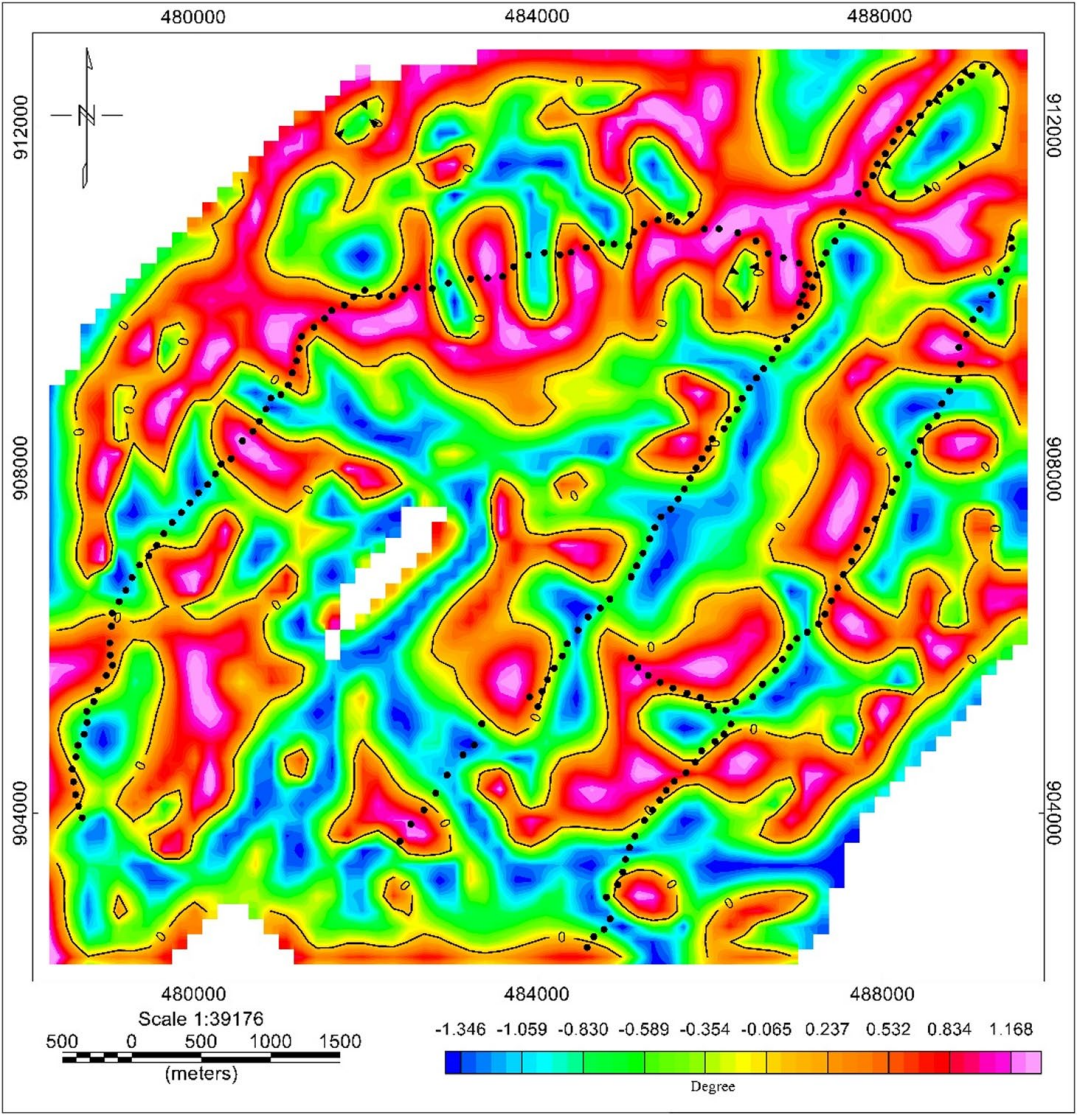
Tilt derivative of Meki-Alemtena area.

From tilt derivative map, the location of faults and different contacts was simply detected. Tilt-derivative filtering (Fig. 16) reveals structural contacts with high clarity. Zero-contour lines mark boundaries between magnetic source bodies, showing: NE–SW faults parallel to the WFB, E–W transfer faults near the watershed divide, and N–S structures that may act as secondary conduits/barriers. It reflects the position of strong gradients at the contact boundaries of magnetic sources where magnetic susceptibilities between positive and negative anomalies abruptly shift. These structural trends agree with known MER tectonics^29^ and match VES-derived anisotropy variations. The E–W trend aligns closely with the surface watershed boundary and likely represents a subsurface hydraulic transition zone, a key novel finding of this study.

### Geological interpretation of the structure

**Fig. 17:**
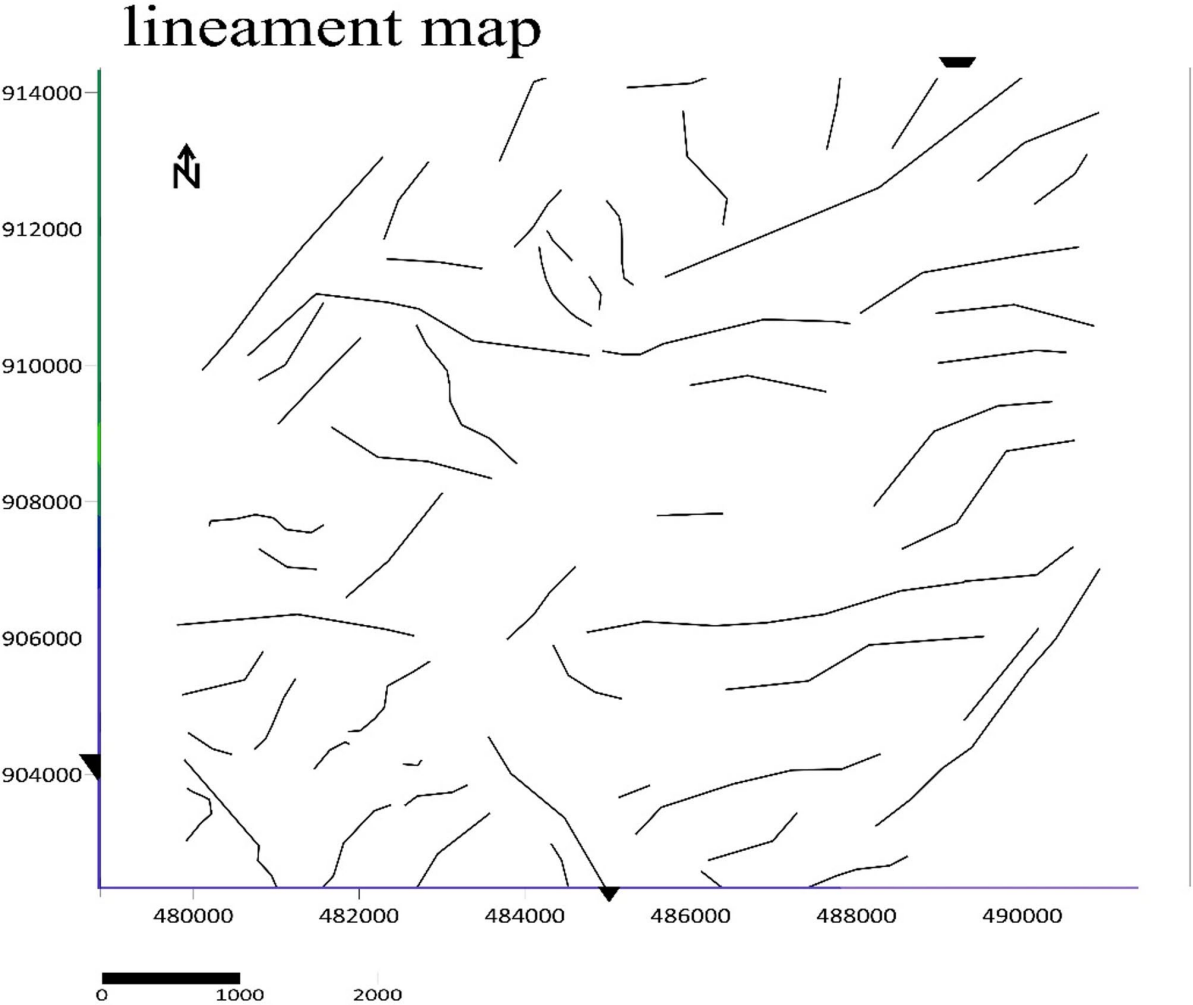
Lineament map of Meki–Alemtena area.

The interpretation of structural map has been drawn from tilt derivative and Analytic signal map to highlight the different lineaments in the area. This lineament reveals the structural complexity in the basement, characterized by line trends. The analysis of the interpretive structure map shows three dominant structural trends with different intensity and length. The N–S, NE–SW, and E–W are the main trends in the studied area as shown in (Fig. 17). The relationships among these three trends suggest that the area was subjected to more than a single tectonic event. The E–W trend is approximately along the direction of water divide which separates the waterflow direction in the region in to north and south. Some of the structural trend in the south section of the major trend is along SE direction. It provides an extensive overview of the underground features that could regulate groundwater flow. Those trends at the South or North of the E–W trends are base for the water flow to opposite direction.

### Integration of VES and magnetic survey results

The integration of VES, magnetic, and borehole data followed a structured three-step workflow to link aquifer hydraulic properties with subsurface structural features. VES inversion delineated vertical stratigraphy, while Dar Zarrouk parameters quantified aquifer transmissivity and protective capacity. Magnetic data provided lateral structural information, with strong gradients and high analytic signal amplitudes highlighting fractured ignimbrite units along NE–SW- and E–W-trending faults that influence hydraulic connectivity. Layer models from VES inversion revealed considerable variations in resistivity and layer thicknesses, reflecting the heterogeneous nature of volcanic and sedimentary units. Evaluated together, zones of high transmissivity and hydraulic conductivity, mainly in the northern and northwestern sectors, coincide with structural corridors that serve as primary pathways for groundwater flow, consistent with observations in other rift-related and hard-rock volcanic aquifers^60^.

Magnetic-derived lineaments, extracted using analytical signal and tilt derivative filters, show dominant NE–SW and E–W trends across the study area. These lineaments align with the Wonji fault belt and transverse rift structures, which are recognized as key controls on secondary porosity in volcanic terrains^29^. Their correspondence with zones of elevated transmissivity and resistivity contrasts confirms the influence of tectonic structures on aquifer geometry and hydraulic connectivity^9,58^. These observations, consistent with other volcanic aquifers in the East African Rift and Arabian Shield, demonstrate that combining resistivity, magnetic, and structural analyses provides a robust framework for groundwater resource planning in data-scarce rift environments^31^.

### Limitations and recommendations

To characterize groundwater conditions in the study area, only sixteen VES points were available; this number can be substantially increased in future investigations. Logistical constraints limited the spatial coverage of groundwater inventory and resistivity data, thereby restricting a more comprehensive assessment. The application of improved methodologies, such as the integration of advanced geophysical techniques with water quality analysis, offers significant potential for future research. Such integrated approaches can assist decision-makers in groundwater exploration and management, especially in arid regions such as the Meki–Alemtena area, where groundwater plays a critical role in agricultural, and domestic water supply.

## Conclusions

The integration of Vertical Electrical Sounding (VES) and magnetic survey data has provided significant insights into subsurface structures and groundwater potential in the study area. VES data revealed variations in resistivity that identify groundwater-bearing zones at multiple depths, particularly within thick, low-resistivity horizons favorable for groundwater storage. These zones, primarily found in fractured and weathered units such as alluvial deposits and ignimbrites mixed with sand, indicate promising conditions for aquifer development. The consistency between geophysical survey findings and borehole lithology further validates these interpretations. The Dar Zarrouk parameter was instrumental in assessing aquifer characteristics, particularly salinity and protective capacity. While most aquifers exhibited saline qualities, freshwater zones were identified in southeastern sections, with varying degrees of aquifer protection based on longitudinal conductance values. Magnetic survey data, including tilt derivative analysis, identified E-W and NE-SW structural trends that guide groundwater flow in the area. Localized faults and fractures observed in resistivity and magnetic data underscore their essential role in facilitating groundwater movement through permeable pathways. To deepen the understanding of groundwater flow and structural controls in the area, further studies should integrate other geophysical data with regional hydrogeology, geology, and structural geology. Furthermore, detailed structural geological surveys should examine fracture and fault orientations, including strike, dip, and extension, to provide a comprehensive understanding of subsurface hydrogeology. The use of higher-resolution geophysical techniques will also improve the detection of small-scale subsurface features, aiding effective groundwater management. Moreover, this research contributes to the broader understanding of groundwater dynamics in rift valley settings, providing a methodological framework that can be applied to similar regions within Ethiopia and beyond.

## Data Availability

The data presented in this study are available upon request from the corresponding author.
